# The cost of tsetse control using ‘Tiny Targets’ in the sleeping sickness endemic forest area of Bonon in Côte d’Ivoire: Implications for comparing costs across different settings

**DOI:** 10.1371/journal.pntd.0010033

**Published:** 2022-01-05

**Authors:** Fabrice Courtin, Dramane Kaba, Jean-Baptiste Rayaisse, Philippe Solano, Steve J. Torr, Alexandra P. M. Shaw

**Affiliations:** 1 Institut Pierre Richet (IPR), Institut National de Santé Publique (INSP), Laboratoire Mixte International sur les Maladies à Vecteurs, Bouaké, Côte d’Ivoire; 2 Institut de Recherche pour le Développement (IRD) UMR 177 Intertryp IRD-CIRAD, Université Montpellier, Montpellier, France; 3 Centre International de Recherche-Développement sur l’Elevage en zone Subhumide (CIRDES), Bobo-Dioulasso, Burkina Faso; 4 Liverpool School of Tropical Medicine (LSTM), Liverpool, Merseyside, United Kingdom; 5 Division of Infection and Pathway Medicine, Deanery of Biomedical Sciences, College of Medicine and Veterinary Medicine, The University of Edinburgh, Edinburgh, United Kingdom; 6 AP Consultants, Walworth Enterprise Centre, Andover, United Kingdom; Makerere University, UGANDA

## Abstract

**Background:**

Work to control the gambiense form of human African trypanosomiasis (gHAT), or sleeping sickness, is now directed towards ending transmission of the parasite by 2030. In order to supplement gHAT case-finding and treatment, since 2011 tsetse control has been implemented using Tiny Targets in a number of gHAT foci. As this intervention is extended to new foci, it is vital to understand the costs involved. Costs have already been analysed for the foci of Arua in Uganda and Mandoul in Chad. This paper examines the costs of controlling *Glossina palpalis palpalis* in the focus of Bonon in Côte d’Ivoire from 2016 to 2017.

**Methodology/Principal findings:**

Some 2000 targets were placed throughout the main gHAT transmission area of 130 km^2^ at a density of 14.9 per km^2^. The average annual cost was USD 0.5 per person protected, USD 31.6 per target deployed of which 12% was the cost of the target itself, or USD 471.2 per km^2^ protected. Broken down by activity, 54% was for deployment and maintenance of targets, 34% for tsetse surveys/monitoring and 12% for sensitising populations.

**Conclusions/Significance:**

The cost of tsetse control per km^2^ of the gHAT focus protected in Bonon was more expensive than in Chad or Uganda, while the cost per km^2^ treated, that is the area where the targets were actually deployed, was cheaper. Per person protected, the Bonon cost fell between the two, with Uganda cheaper and Chad more expensive. In Bonon, targets were deployed throughout the protected area, because *G*. *p*. *palpalis* was present everywhere, whereas in Chad and Uganda *G*. *fuscipes fuscipes* was found only the riverine fringing vegetation. Thus, differences between gHAT foci, in terms of tsetse ecology and human geography, impact on the cost-effectiveness of tsetse control. It also demonstrates the need to take into account both the area treated and protected alongside other impact indicators, such as the cost per person protected.

## Author summary

Sleeping sickness is a fatal disease in Africa caused by trypanosomes transmitted by the bite of infected tsetse flies. The World Health Organization has set the absence of new infections as a goal for 2030. To achieve this, screening and treatment of patients is supplemented by tsetse control. Tiny Targets are small insecticide-impregnated panels of blue cloth and black netting which attract and kill tsetse. To maintain the momentum in these last stages of eliminating the disease, it is important to understand the costs of vector control. This paper presents the results of two years’ cost monitoring in Bonon, an area covering 130 km^2^, home to 120,000 people. Since 2016, ~2,000 Tiny Targets have been deployed annually at a cost of USD 471 per km^2^ per year. Bonon is a degraded forested area and a relatively high number of targets was required as tsetse are present throughout. Nevertheless, the cost comes to only USD 0.5 per person protected per year. Compared to operations using Tiny Targets in Uganda and Chad, the cost was higher per km^2^ protected, reflecting the need to place targets throughout the area, but low per person protected compared to Chad, highlighting the need to use a range of comparators.

## Introduction

In 2007, a consultation held at the World Health Organization (WHO) headquarters concluded that the elimination of the gambiense form of human African trypanosomiasis (gHAT) as a public health problem was a viable goal and the year 2020 was set as the target for achieving this [1,2]. In 2012 this was reviewed and the goal of elimination, defined as the absence of transmission resulting in zero cases reported, was set for 2030 [3]. The chronic form, gHAT, caused by *Trypanosoma brucei gambiense* is found largely in West and Central Africa and also in limited parts of South Sudan and north-western Uganda in East Africa. The acute form (rHAT), caused by *Trypanosoma brucei rhodesiense* is found in East Africa. Both forms of the disease are normally fatal in untreated individuals. Control of gHAT has relied mainly on active disease surveillance through periodic screening programmes, followed by treatment of patients found and sometimes supplemented with tsetse control [4]. Both forms of the disease, also known as sleeping sickness, have been known for centuries and chronicled by colonial doctors and historians [5–7].

Throughout Africa, large-scale programmes to find and treat infected people were implemented and by the mid-1960s the disease was considered to have been successfully controlled [8]. However, by the late 1990s is was obvious that a major resurgence of the disease had occurred, linked to a dramatic decline in medical surveillance from the early 1970s onwards and to profound environmental changes as people and livestock moved into new areas, affecting not just historical foci but also giving rise to new foci of gHAT and rHAT. The number of reported cases in Africa had risen to nearly 40,000 by 1998, close to the peak number found at the beginning of the 1940s [9], and the WHO estimated the true number of infected individuals to be around 300,000–500,000. Since then, intensive screen-and-treat programmes, again supplemented with vector control in some foci, have already reduced the number of reported cases below the threshold of 2,000 cases a year set by WHO as the level where the disease would be eliminated as a public health problem. However, the threshold of one or fewer cases per 10,000 people has not been met in all locations [10]. The second goal set out [3], of a cessation of transmission, and thus no new infections by 2030, seems achievable. Here, once again vector control has a key role to play, following on from its contribution to the elimination of gHAT as a public health problem [11].

Vector control has been used since the early 20^th^ century to supplement case finding and treatment of both gHAT and rHAT and, in particular, to bring epidemics under control and sometimes aiming at elimination. On the Island of Principe, where sleeping sickness was a major problem, tsetse were successfully eliminated between 1910 and 1914 [12–14]. In West Africa, a number of projects have been undertaken. Notably, from 1955–1970, ground spraying in north-eastern Nigeria eliminated tsetse from one of the country’s two primordial gHAT foci, as classified by Duggan [14]. This consisted mainly of *Glossina tachinoides* and *G*. *p*. *palpalis* alongside some pockets of *G*. *morsitans submorsitans*, located along the rivers flowing into the Lake Chad basin. This gHAT focus was thus permanently eliminated [15–17]. In Côte d’Ivoire early initiatives to control tsetse in gHAT foci included the use of residual insecticides in the Abengourou and Daloa forest areas [18] followed by the use of blue insecticide-treated targets (‘screens’) and ground spraying at the end of the 1970s, and aerial spraying using helicopters in the Bouaflé area in 1978–79 [19]. After some years working with traps, the use of insecticide-treated blue screens was first trialled in 1981 in Burkina Faso along the Léraba River, followed by a pilot programme in Vavoua [20–22]. In the latter focus, local planters were given screens to place around their plantations, leading to a community-based tsetse control programme in Vavoua, undertaken from 1987–1990 [22]. This included a detailed assessment of costs. Nearly 40,000 screens were deployed protecting an area of about 1,500 km^2^ and 25,000 people. Nearly a decade later, vector control took place from 1995–1997 in the hyperendemic gHAT focus of Sinfra, with 13,000 screens being distributed to local planters, supplemented by over 200 insecticide-impregnated traps [23]. During this period, tsetse control was also undertaken in the north of Côte d’Ivoire, near Korhogo, in this case to control animal trypanosomiasis and to support cattle production in that area. The method was low density trapping (0.3 traps per km^2^ protected). Trials began in the late 1970s and by 1993 an area of nearly 51,000 km^2^ was protected [24,25] and its costs were also assessed [26].

Tsetse control in the peri-urban gHAT focus of Bonon began in 2016, with the deployment of nearly 2000 Tiny Targets in an area of 130 km^2^ including Bonon town and the rural area to its south [27,28]. Here we report on the costs of this intervention. The work in the Bonon focus is the third Tiny Target project for which the full intervention costs have been assessed, the others being in Uganda (2012–2013) and Chad (2015–2016) [29,30]. The results from all three cost studies are compared and the implications of the differences in costs in relation to a range of metrics are discussed.

## Methods

### Ethics statement

Ethical clearance for this work was granted by the Comité National d’Ethique de la Recherche (CNER) of the Ministère de la Santé et de l’Hygiène Publique—Côte d’Ivoire. Approval reference number: 030-18/MSHP/CNER-kp.

### Study area

**Fig 1:**
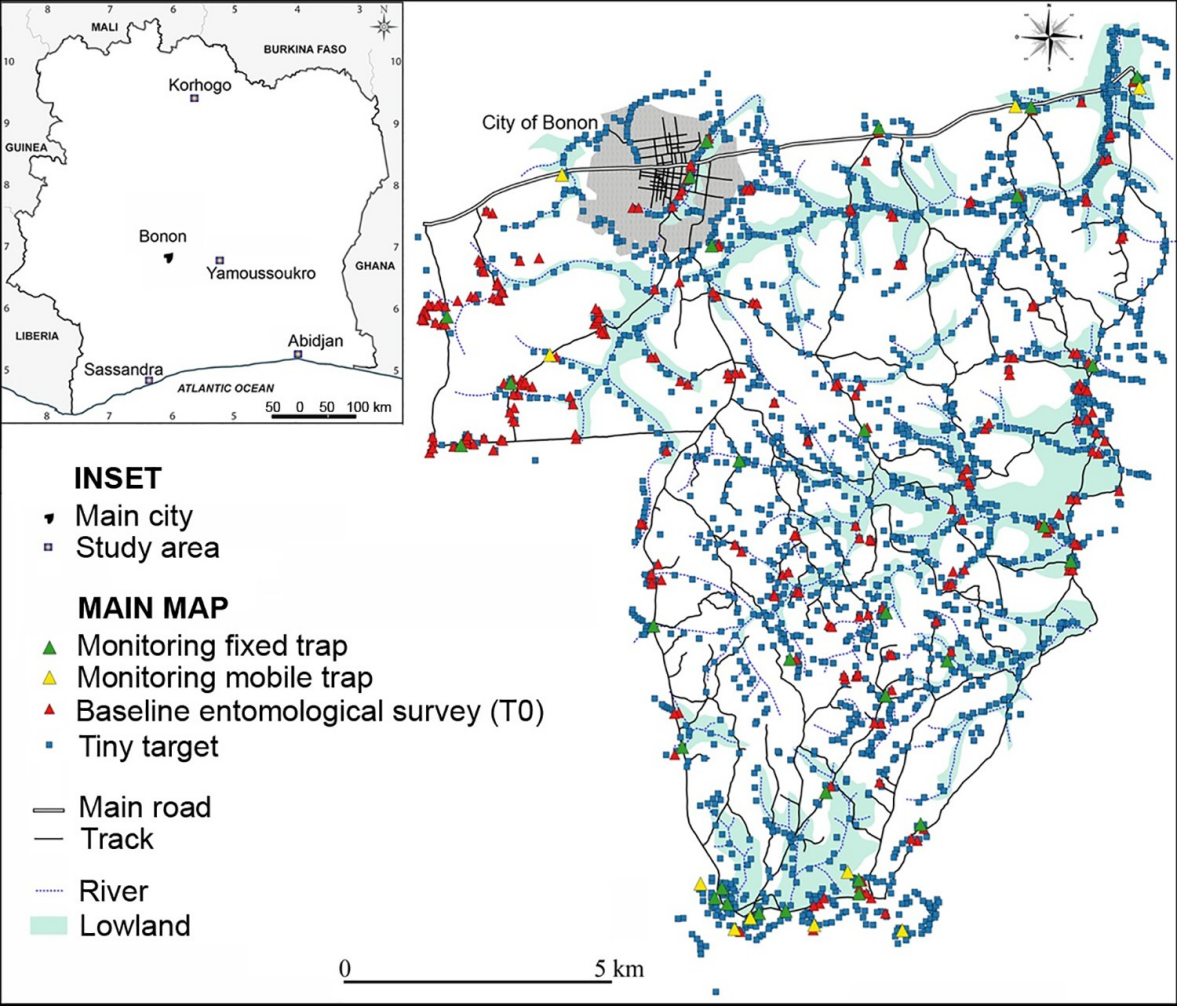
The Bonon gHAT focus showing target deployment and location of monitoring traps 2016–2017. Inset: Côte d’Ivoire showing location of Bonon town.

Bonon is a town located in the Marahoué region, about 100 km west of Yamoussoukro, the political capital of Côte d’Ivoire (Fig 1). Bonon town and the rural area to the south of it, where tsetse control has been implemented, have a population of some 120,000 people. The main livestock kept are pigs and cattle, with a population of 2471 pigs and 1710 cattle recorded [27]. Bonon lies in the Upper Guinean forest zone with much low-lying ground which is seasonally flooded (see Fig 1). Cash (coffee, cocoa, cashew tree) and food crops (maize, cassava, banana, rice) have gradually replaced the natural forest in the area. Bonon was first identified as a gHAT focus in the 1970s [31]. Analyses of gHAT cases from the focus showed that some patients lived in Bonon but travelled frequently to rice, cassava and maize fields immediately south of the town while others travelled further south to their cocoa and coffee plantations [32]. These findings were similar to those observed previously [22]. For Bonon, further analysis of the cohort of patients, of whom 75% lived in the town, along with entomological studies in the focus, suggested that urban transmission by *G*. *p*. *palpalis* feeding on people occurs [33]. The boundary of the tsetse control intervention area was therefore designed to protect people in both the urban and rural settings identified [34], and also took into account the river and transport networks.

### Data collection

In 2015, cost data were produced during field missions for the baseline tsetse survey (designated as T0) and the preliminary sensitization of the human population was undertaken. Cost data were also collected and compiled after each of the main tsetse control activities carried out in the years 2016 and 2017 (sensitisation, target deployment, tsetse monitoring, target maintenance). The data production protocol was modified from that originally trialled in Uganda [29]. The new protocol was applied contemporaneously in both Chad and Côte d’Ivoire. This protocol and the blank Microsoft Excel file on which it is based are available as additional files alongside a completed spreadsheet for Chad [30] and the completed field trip cost spreadsheet for Côte d’Ivoire is available as S1 Data. The modalities for each field trip were given in a travel authorisation from the Institut de Recherche pour le Développement (IRD). The cost of activities was worked out after every trip, looking at the list of receipts and calculating the time spent in the field by the team.

The data compiled routinely during and after each mission covered:

- length and purpose of mission, achievements (e.g. number of targets deployed, traps monitored, sensitisation meetings held);
- staff supervising and participating in the mission, per diems paid and days worked, hiring and payment of local labour or involvement of community health and other workers;
- vehicle use, itineraries, fuel costs, spares and other maintenance costs paid for during the trip;
- expenditure on equipment and consumables such as traps, targets, protective clothing, telephone and GPS batteries.

To produce a comprehensive cost figure for the Tiny Target work, a share of staff salaries, vehicle depreciation and overheads, as well as the cost of administering the project, needed to be added to these field costs, as explained below.

### Economic analysis of costs

#### Full cost approach

There are several levels at which the costs of any operation can be analysed. These can broadly be divided into either: (i) financial analyses, which monitor the costs to different stakeholders and funders (donors, ministries, research institutes, community organisations or individuals such as planters or livestock keepers) or (ii) economic analyses, which seek to include all of the costs to society arising from a particular operation. In the case of the Tiny Target programme, the objective of this paper is to look at the economic cost of the work. The analysis thus needs to cover inputs from the various organisations involved.

The notion of a ‘full cost’ approach was developed to distinguish a comprehensive calculation of tsetse control cost from one in which only core expenditures (targets, traps, insecticide, flying time) are quantified [35,36].

#### Research and control components

Articles on tsetse control operations have often contained a research component, as techniques are being developed and trialled in different locations. In this analysis, as in earlier work [29,30,37], the costs have been adapted to remove the research component. For this study to be consistent with the others, this has meant leaving out the laboratory materials and extra time required for tsetse dissection and analysis and standardising salaries and per diems at the level paid by the Institut Pierre Richet (IPR) to its research staff and students, as relevant.

#### Prices

As explained above, costs were recorded as they were incurred. The prices of key items remained very constant during the two years studied, 2016 and 2017, with regular differences occurring, such as fuel being more expensive outside the towns. To be consistent with the Chad study [30], here the 2016 price levels were retained where there was a specific price change. This occurred only in the case of field travel allowances. Expenditures were mostly in West African BCEAO (Banque Centrale des États de l’Afrique de l’Ouest) CFA (Communauté Financière Africaine) Francs (XOF) with some items (targets, customs fees) in United States dollars (USD) or Euros (EUR). The CFA franc is pegged to the Euro at a rate of 1 EUR = 656 F CFA. All monetary amounts were converted to USD at 1 USD = 593 F CFA, the rate applying for the calendar year 2016. Where USD prices from other studies are cited, these were converted to USD using the exchange rate applying at the time when they were incurred and then to 2016 price levels using the mid-year US inflation rate https://inflationdata.com/inflation/Inflation_Rate/HistoricalInflation.aspx [36].

Since 2020, Vestergaard Ltd has been supplying Tiny Targets at zero cost to the gHAT elimination programme. During the period covered by this study, Tiny Targets were purchased from Vestergaard at a cost of USD 2.50 each. This is the cost included here, not just because it reflects the price paid, but, because in an economic, as against a financial analysis, where an item’s cost is subsidized, its full cost to society should nevertheless be the one included.

Price levels vary from country to country and a method exists for converting prices to an international standard level by adjusting for purchasing power parity (PPP) to convert costs to so called ‘international dollars’. However, to date all tsetse and trypanosomiasis control costs have been presented using so called ‘nominal prices’ which are the local, in-country, market prices [36]. This paper follows that convention by using nominal prices so that the costs presented provide an easily interpreted estimate. If wanted, these can be adjusted to reflect prices in other countries.

#### Capital items and vehicles

Durable goods with a useful life of more than one year are classified as capital items. This means that their value has to be spread over the years during which they are in use, whether for the activity being costed, or for another purpose. Furthermore, these items are very likely to be used for other activities undertaken by the organisations involved in the Tiny Target work, so only a proportion of their annual depreciation was allocated to the Tiny Targets project. Global positioning system (GPS) sets were costed on the basis of a five year useful life with half of their use being for the Tiny Targets work. They were not used for sensitisation, so their depreciation was divided between the other activities in line with the number of sets used and the duration of field trips. Targets were replaced annually, so were not included in capital items. Traps, on the other hand, were conservatively estimated to be usable for 12 field trips. The costs of some other relatively durable items, such as machetes, were included in full when purchased, as in practice their useful life was usually a year. For the administrative support, the cost of a computer, printer, scanner, voltage stabiliser and extension leads were all depreciated over three years.

Vehicles used for fieldwork consisted of four-wheel drive (4x4) field vehicles (usually a Toyota Land Cruiser) and three Yamaha motorbikes. In line with project experience, all vehicles were assumed to have a six-year useful life. Estimates were also made of the project’s component of the costs of insurance, road tax, servicing and road-worthiness checking of shared vehicles. These costs were paid by the organisation owning the vehicles. They were apportioned according to the proportion of annual kilometres travelled for the Tiny Targets work, and the total annual amounts (rounded to USD 1800) were allocated between the different project activities according to the kilometres travelled for each one.

#### Staff and Project administration

The Excel personnel cost sheet provided a detailed breakdown of the time that staff from each of the three institutes spent in the field. To this were added days that staff spent working on the project at their base before and after field trips. These ranged between 1.5 and 2.5 days per person per trip, with two months’ total time per year costed for the project coordinator. Project administration costs were calculated for each institute, based on a share of the time of a secretary and accountant: 0.5% for the Centre International de Recherche-Développement sur l’Elevage en zone Subhumide (CIRDES) 1.5% for the IPR and the IRD. As was done for the capital items, a share of the annual administration cost was allocated to each activity in proportion to the number of field days it involved.

### Comparison with the costs of Tiny Target operations in Chad and Uganda

This study completes the series of three on the costs of Tiny Target operations [29,30]. To understand how different tsetse bioecologies, human geography and organisational approaches affect costs, the interventions were compared. To do this, the prices for Uganda were updated to 2016 levels as explained above. In the Uganda cost paper [29] administration and office costs were assigned a separate category. For consistency with the approach used in Chad and Côte d’Ivoire, they were extracted and allocated to the different project activities (target deployment, tsetse monitoring, target maintenance, sensitisation) in proportion to the duration of each activity. A range of metrics were used to compare the costs for the three locations. These were either linked to the operation itself: annual cost of the operation per target deployed, target density and the area where they were deployed, traditionally described as the ‘treated area’ or to the operation’s potential impact: the area of the gHAT focus protected from tsetse and the number of people in that area.

## Results

### Timing of activities

**Fig 2:**
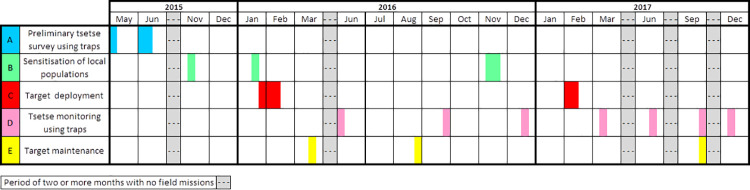
Schedule of work for which costs were collected and analysed.

The Tiny Target control programme involved five distinct activities, as outlined in Fig 2 and Table 1. Work began with a detailed tsetse survey which was planned in April-May 2015 and was carried out in June 2015, setting the ‘T0’ baseline. The other preliminary activity was the production of a radio spot for informing local people about the Tiny Target project and its objective, followed by focus group meetings, undertaken at the end of 2015, prior to the first deployment of targets in January 2016. From then on the project settled into a steady routine. Target deployment occurred at the start of the year followed by tsetse monitoring missions using traps every three months, numbered consecutively T1, T2, etc. There were only three monitoring missions in 2016. These were interspersed with one or two target maintenance sessions, checking on the state of targets, adjusting their number (in the first year) and picking up fallen targets. The initial sensitisation was undertaken in November 2015 and repeated just before the first deployment in 2016 and again at the end of that year, before the 2017 deployment. The Tiny Target operation continued at full strength through to 2019, with some reduction in activities in 2020.

**Table 1:** Timing and duration of field activities.

Activity	Timing	Duration (days)	Description
Preliminary tsetse survey preparation	May 2015	3	Meeting to decide on protocol, organise roles and cooperation between teams
Preliminary tsetse survey T0 baseline	June 2015	12	Field trip, sampling 278 sites using 120 monoconical traps
Production of sensitisation materials	Nov 2015	3	Creation of radio spot for sensitising populations
Initial sensitisation	Jan 2016	7	7,000 people directly contacted in the course of 45 focus group meetings
First target deployment	Jan-Feb 2016	22	1,880 targets deployed
Target maintenance	March 2016	7	Targets checked and relocated as necessary in relation to flooding
Trap monitoring T1	May-June 2016	9	Traps deployed at 40 sites
Target maintenance	Aug-Sept 2016	7	Targets checked, repositioned and 30 more deployed
Trap monitoring T2	Sept-Oct 2016	9	Traps deployed at 40 sites
Re- Sensitisation	Nov 2016	12	Sensitisation of communities
Trap monitoring T3	Dec 2016	10	Traps deployed at 40 sites
Target redeployment	Jan-Feb 2017	17	1,997 targets replaced
Trap monitoring T4	March 2017	9	Traps deployed at 40 sites
Trap monitoring T5	June 2017	8	Traps deployed at 40 sites
Trap monitoring T6	Sept 2017	9	Traps deployed at 40 sites
Target check	Sept 2017	9	Placement and condition of deployed targets checked
Trap monitoring T7	Dec 2017	8	Traps deployed at 40 sites

### Human resources and organisational structure

The IRD and IPR teams based in Bouaké managed the programme and provided most of the staff, while CIRDES in Bobo-Dioulasso gave support when needed, especially at the beginning of the programme (baseline entomological survey), by supplying vehicles, as well as inputs for the research part of the programme. Research inputs and oversight were also provided by the Liverpool School of Tropical Medicine (LSTM). Field trips included two or three researchers, two to four IPR technicians and up to six IPR students as well as drivers.

The time invested in the control operation was considerable. Table 2 gives the number of person field days for each year and the number of staff employed on each mission. When the person non-field days for preparing and processing the field trips, calculated as explained in the Methods section, are added, the total person days come just under 650 each for 2016 and 2017. The non-field days thus add about a third to the total.

**Table 2:** Field travel days: staff from the research institutes.

Activity	2015		2016		2017	
	Number of staff	Person days	Number of staff	Person days	Number of staff	Person Days
Tsetse surveys^a^ and monitoring^**b**^	15	175	6	162	4–5	156
Sensitisation	–	–	6	42	6	72
Deployment	–	–	11	235	7	204
Maintenance	–	–	3–7	70	3	27
**Totals**	–	**175**	–	**509**	–	**459**

Notes

^a^Preliminary tsetse survey (T0) undertaken before target deployment.

^b^There were three monitoring survey rounds undertaken in 2016 and four in 2017.

The work in the field relied on inputs from district health service workers from Bonon, a doctor and a nurse (district medical officer–DMO and a state nurse–SN). We also involved community health workers (CHWs) belonging to each village of the tsetse control intervention area. One worker from the district livestock services also participated, especially during the sensitisation campaigns. These individuals were involved from the beginning of the programme and were paid an allowance every three months for their work monitoring targets and sensitising local populations plus per diems during the IPR field missions in which they participated. The project was also voluntarily supported by community heads, religious leaders and school teachers who reinforced the sensitization campaign.

### Baseline tsetse survey and monitoring

The baseline tsetse survey (T0) was undertaken in 2015 over a period of 12 days with 15 people. Monoconical Vavoua traps for sampling tsetse were placed at 278 locations (Table 1 and Fig 1). The catch of tsetse was recorded and a subset of the flies was examined microscopically for presence of trypanosomes. No molecular tests were done to confirm the trypanosome species. Following this, seven monitoring rounds (T1 to T7) were undertaken during the period in which costs were collected: three in 2016 and four in 2017. The monitoring rounds typically lasted nine days and the average number of staff was six in 2016 and varied between four and five in 2017 (Table 2).

For routine monitoring, sentinel traps were placed at 40 sites of which 30 were fixed and 10 mobile. The 30 fixed traps were used for the monitoring of the changes in tsetse fly densities. The mobile traps were set up to check the situation in the intervention area not covered by the 30 fixed to identify areas with high numbers of tsetse outside the intervention area. All fixed and mobile traps were set up for 48 hours during each monitoring session. For the baseline T0 survey only 4x4 motor vehicles were used, thereafter motorcycles were used as well. The T0 survey cost just over USD 13,000 working out at just over USD 100 per km^2^ protected. Thereafter the average cost of monitoring rounds came to almost exactly USD 5,000 (Table 3) or USD 38 per km^2^ protected.

**Table 3:** Cost of baseline tsetse survey and subsequent monitoring using traps.

Item (percentage of two year’s total monitoring costs)	2015 Baseline tsetse survey^a^ USD	2016 Monitoring USD	2017 Monitoring USD	Average cost per Monitoring round^b^ USD
**Specialised equipment (0.5%)**				
Traps (depreciation)^c^	112	70	93	23
Trap cones, metal supports, stakes and sleeves	10	6	8	2
**Transport (13.1%)**				
Share of four wheel drive (4x4) vehicle overheads	450	290	455	106
4x4 vehicle maintenance and repairs	128	641	185	118
Share of motorcycle overheads	–	495	555	150
Motorcycle maintenance and repairs	–	186	93	40
Fuel	329	781	911	242
**Staff (68.2%)**				
Share of staff salaries	5,223	6,606	6,294	1,843
Travel allowances	5,970	5,160	5,818	1,568
**Community workers (13.1%)**				
Payment to CHWs, DMO and SN^d^	172	1,669	2,926	657
**Administrative support (2.3%)**	261	385	399	112
**Consumables and equipment (2.8%)**				
GPS sets (depreciation)	44	87	87	25
Protective clothing, hammers, machetes, pliers, wire, lime, grease, etc.^e^	193	19	161	26
Sundries, including GPS batteries, internet, telephone and stationery	190	323	302	89
**Total**	13,082	16,718	18,287	5,001
**Cost per km**^**2**^ **protected**	100.6	128.6	140.7	38.5

Notes

^a^ The T0 cost was treated as a capital item in the overall cost summary table. Its cost was spread over 4 years of full scale target deployment (2016–2019). Since the start of 2020 work has continued with fewer targets deployed.

^b^There were 7 rounds over the 2 years.

^c^30 traps were used for regular monitoring and 120 for the baseline tsetse survey.

^d^ CHWs–Community Health Workers, DMO—district medical officer, SN–state nurse.

^e^Protective clothing consisted of boots, raincoats and mosquito nets.

The cost of depreciation on traps was only 0.5% of the total, whereas two thirds of costs were for staff salaries and per diems. The cost per trap for the trap fabric was USD 6.50, supplied by Vestergaard Ltd. To this was added UD 0.81 for fixings: metal supports, stakes and cloth sleeves to cover the cones which catch the flies. Insurance and freight for the journey from Vietnam to the port of Abidjan in Côte d’Ivoire added 11% to the cost of the fabric. An unexpected customs bill added a further 18%. This was much reduced in subsequent importations, but was included here to illustrate the type of extra expenditures that can arise. The cost of monitoring rounds fell slightly after the early rounds, as experience made it possible to work round the trap sites more quickly.

### Sensitisation

**Fig 3:**
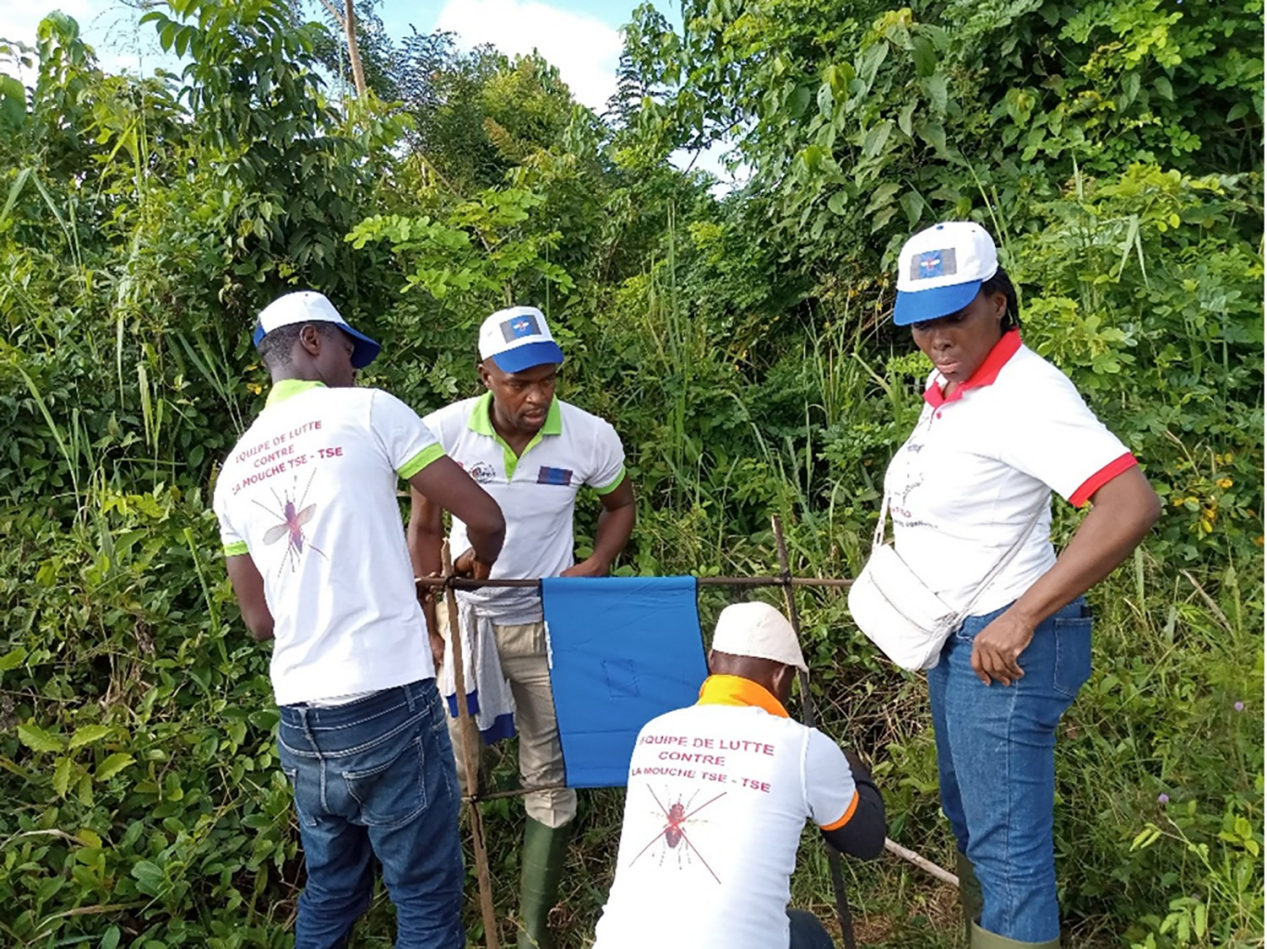
A Tiny Target deployed at the relic forest/human settlement interface.

The sensitisation work began at the end of 2015, with the production of a radio spot for broadcasting in the different languages spoken in the intervention area (French, Gouro, Malinke, Baoulé, Lobiri, Malinke). This broadcast explained the ecology of tsetse flies, the human and animal diseases they transmit and the purpose of Tiny Targets. Sensitisation activities undertaken in January 2016 contacted some 7,000 people via 26 schools, 17 religious leaders, 42 village heads and 14 local societies. A comic book [38] was printed and distributed to teachers, religious leaders, village heads and local societies. A specially produced T-shirt, showing a Tiny Target on the front and the name of the team “Equipe de lutte contre la mouche tsé-tsé” on the back, was worn by team members (local health workers, IPR, IRD) in the field (Fig 3). Then, in 2017, the responsibility for sensitisation was transferred to the local communities. The initial results of the target operation, in terms of reduction in tsetse fly density, were shared with the communities. The costs of sensitisation are summarised in Table 4.

**Table 4:** Cost of producing sensitisation materials in 2015 and operational missions in 2016 and 2017.

Item (average percentage of two years’ total costs)	2016 USD	2017 USD
**Specialised equipment (19.0%)**		
Cost of creating radio broadcast^a^	126	239
Cost of broadcasting on radio	674	337
Printing of sensitisation materials (comic book)	422	84
Megaphones, T-shirts and caps	246	573
**Transport (9.7%)**		
Share of 4x4 vehicle overheads	190	105
Maintenance of 4x4 vehicle	–	85
Share of motorcycle overheads	160	140
Fuel	273	422
**Staff (60.9%)**		
Share of staff salaries	1,766	2,820
Travel allowances	1,653	2,428
**Community workers (4.7%)**		
Payment to health district staff	422	253
**Administrative support (2.1%)**	118	174
**Consumables and equipment**^**b**^ **(3.6%)**		
Stationery	259	90
Telephone communications	82	79
**Total**	6,391	7,829
**Cost per km**^**2**^ **protected**	49.2	60.2

Note

^a^ The initial broadcast cost USD 506 to produce in 2015 and was treated as a capital item whose cost was spread over 4 years of full scale target deployment (2016–2019) and the subsequent broadcast improvement in 2017 which cost USD 337 was spread over the 3 remaining years.

### Deployment

Deployment took place during the dry season, spanning late January and early February. The initial deployment in 2016 took 22 field days with a team of 11 people and 1886 targets deployed. Once the trap locations had been decided on and the people working had established a routine, the time required for the activity could be reduced. Thus, the 2017 second deployment of 1,997 targets took seven people only 17 days supported by increased inputs from the community workers (Tables 1 and 2). The increase in the number of targets consisted of an extra 27 deployed during the maintenance activities in August 2016 which were reinforced with an additional 84 targets during the 2017 deployment (Fig 1). The deployment activity is illustrated in Fig 3.

The insecticide-impregnated target cloth and netting were bought from Vestergaard Ltd at a cost of USD 2.50 per target. The Tiny Targets used in Côte d’Ivoire and also in Guinea were 0.75m wide by 0.5m high [28,39] and thus larger than those used in Chad and Uganda [40,41]. They were shipped to Abidjan from Vietnam by air, with insurance and freight adding USD 0.41 to the fabric cost. Two wooden sticks are ‘planted’ in the ground, the target is then attached to these by strings and supported by two further horizontal sticks (Fig 3). These sticks were produced for a lump sum payment by local labourers and added USD 0.14 to the cost of the fabric. As explained above for the traps, in 2016 a further high cost for customs and storage was incurred, which added a further USD 0.69 to the cost of the fabrics. Thus the overall cost per target deployed came to USD 3.74. Taken as a whole, the deployment activity cost an average of USD 215 per km^2^ protected. Targets accounted for a little over a quarter of costs and staff for 60% (Table 5).

**Table 5:** Cost of deploying targets.

Item (percentage of two years’ total costs)	2016 USD	2017 USD
**Specialised equipment (26.0%)**		
Targets	4,715	4,993
Target insurance and freight	773	819
Target customs and storage	1,301	1,378
Production of wooden sticks for target mounting	270	270
**Transport (4.6%)**		
Share of 4x4 vehicle overheads	230	230
4x4 vehicle maintenance and repairs	340	101
Share of motorcycle overheads	190	160
Motorcycle maintenance and repairs	25	51
Fuel	675	590
**Staff (59.7%)**		
Share of staff salaries	8,457	7,993
Travel allowances	9,418	7,454
**Community workers (4.4%)**		
Payment to health district (IE, MCD) and CHW	1,012	1,416
**Administrative support (1.9%)**	554	493
**Consumables and equipment (3.4%)**		
GPS sets (depreciation)	55	55
Telephone and stationery (printing, GPS batteries, anti-virus programme, ink cartridges, paper, etc.)	232	700
Protective clothing and hardware items (boots, rucksack, hammers, machetes, extension cord, tarpaulin)	212	621
**Total**	28,459	27,324
**Cost per km**^**2**^ **protected**	218.9	210.2

### Target maintenance

Target maintenance rounds were undertaken twice in 2016 and once in 2017. During the first round in March 2016, some 400 targets were checked and 76 which were found to be at risk of being carried away by floods were relocated. The community health workers were intensively involved in target maintenance rounds, so as to encourage them to contribute to ongoing target surveillance. During the second round, in August 2016, about 900 targets were visited and moved or set upright again if necessary, which was needed for some 200 targets. An extra 27 targets were deployed in order to reinforce the vector control in areas identified as epidemiological hot spots. Then, in September 2017, 1,257 targets (63% of those deployed) were inspected and their state assessed. Again, any targets that had fallen down were set upright again. It was decided that a second maintenance round was not required in 2017. The costs are shown in Table 6. The average cost per maintenance round was USD 3,639, with the 2016 rounds being significantly more expensive. Maintenance accounted for USD 64 per km^2^ protected in 2016, falling to USD 20 in 2017.

**Table 6:** Costs of target maintenance.

Item (percentage of two years’ total costs)	2016 USD	2017 USD
**Specialised equipment (0.6%)**		
Target replacement (including freight, insurance, customs and storage)	97	–
**Transport (12.7%)**		
Share of 4x4 vehicle overheads	190	110
4x4 vehicle maintenance and repairs	76	–
Share of motorcycle overheads	55	45
Motorcycle maintenance and repairs	–	17
Fuel	557	219
**Staff (67.0%)**		
Share of staff salaries	2,850	898
Travel allowances	2,597	911
**Community workers (14.2%)**		
Payment to health district staff (DMO, SN) and CHWs	1,401	303
**Administrative support (1.8%)**	96	65
**Consumables and equipment (3.7%)**		
GPS sets (depreciation)	33	33
Hardware (hammer, machete, chisel)	84	–
Stationery and telephone	287	34
**Total**	8,323	2,635
**Cost per km**^**2**^ **protected**	64.0	20.3

### Cost summary

The costs of the different activities are summarised in Table 7. Costs are presented as the average for one year’s control work, with the costs of sensitisation materials and the baseline tsetse survey being spread over four years of deployment under the project (2016 to 2019). On this basis, the costs per km^2^ protected come to USD 471 per year, of which the bulk is attributed to target deployment (46%) followed by tsetse monitoring (29%).

**Table 7:** Summary of costs for one year’s tsetse control by activity.

Activity	Average per year USD	% of expenditure	USD/ km^2^ protected
Baseline tsetse survey^a^	3,271	5.3	25.2
Tsetse monitoring	17,502	28.6	134.6
Target deployment	27,891	45.5	214.5
Target checking	5,479	8.9	42.1
Sensitisation^a^	7,110	11.6	54.7
Total	**61,253**	**100.0**	**471.2**

Note

^a^ 25% of the cost of the baseline T0 survey and of the creation of the radio broadcast for sensitisation was attributed to each year’s tsetse control operation.

In terms of the breakdown by expenditure category, the main item is expenditure on staff salaries and per diems (64%) followed by specialised equipment (targets, traps and sensitisation materials) as shown in Table 8. The average cost of targets per year for the sewn and insecticide-impregnated fabric and netting as supplied by Vestergaard was USD 4,854 or 8% of total costs. The addition of target transport, import and assembly costs increased the total cost of targets to USD 7,259 or 12% of total costs. The remaining project costs (just under USD 54,000) imply a ‘delivery’ cost per target deployed of USD 28.

**Table 8:** Summary of costs by category of expenditure.

Activity		Average per year USD		% of expenditure	
Specialised equipment		8,778		14.3	
Vehicle costs		5,139		8.4	
Staff salaries		20,149		32.9	
Staff field allowances		19,212		31.4	
Community workers		4,745		7.7	
Administrative support		1,208		2.0	
Consumables and equipment		2,024		3.3	
Total		**61,253**		**100.0**	

### Cost comparisons for three Tiny Target operations

These results from Bonon are compared to those obtained in Chad’s Mandoul focus and Uganda’s Arua focus in Table 9. The entomological aspects of the three operations have been described [28,40,41] as well as for a similar operation in Guinea [39]. The three projects had very different characteristics, both in terms of target deployment and for a range of cost metrics. The implications of these differences are further discussed below.

**Table 9:** Comparison of key indicators for three Tiny Target tsetse control projects in gHAT foci.

Calculation	Chad 2015–2016	Côte d’Ivoire 2016–2017	Uganda^a^ 2012–2013
Average annual cost (USD)	56,133	61,253	21,982
Cost of targets per year (USD)	4,667	7,259	3,269
Total number of targets deployed	2,708	1,939	1551
Target cost as % of total^b^	8.3	11.9	15.3
Cost per single target (USD)^b^	1.56	3.74	1.40^c^
Other costs (USD)	51,466	53,994	18,614
‘Delivery’ cost per target (USD)^d^	19.0	27.9	12.0
Number of km^2^ protected	840	130	250
Targets per km^2^ of area protected	3.2	14.9	6.2^e^
Annual cost per km^2^ protected (USD)	66.8	471.2	88.0
Number of km^2^ treated	45	130	16
Targets per km^2^ treated	60	15	97
Annual cost per km^2^ treated (USD)	1,247	471.2	1,373
Number of people protected	39,000	120,000	100–125,000
People per km^2^ protected	45	920	400–500
Annual cost per person protected (USD)	1.44	0.51	0.18–0.22

Notes

^a^Costs for Uganda have been converted to 2016 price levels in order to be equivalent to those for Chad and Côte d’Ivoire. At the time the costs were estimated, the maintenance round involved replacing 61% (950) of the 1551 targets, and Uganda has since gone on to twice yearly deployments.

^b^The cost per target includes the fabric, netting, fixings, freight and associated costs.

^c^ Note that the slightly lower cost per target for Uganda reflected the calculation of sea freight costs rather than the actual air freight costs as for Chad and in Côte d’Ivoire.

^d^The delivery cost per target is calculated by dividing the ‘other costs’ by the number of targets deployed at any one time. This overestimates the cost in Uganda as there was a partial second deployment.

^e^The target density in the project area costed in 2012/13 [29] was 6.2/km^2^ as against 5.7/km^2^ for the whole project area [40].

## Discussion

In terms of the methodology used to calculate the costs of the operation in Bonon, it should be noted that in the same way as for the Uganda and Chad costings, the purely research elements have been excluded as explained above. Thus some USD 720 of laboratory materials used for dissecting tsetse were not included in the control costs, along with the extra time required in the field during the T0 preliminary survey and on subsequent monitoring rounds and on return to headquarters for processing samples. Adding this would increase costs by just under 2%. Salaries and per diems were priced at the levels paid to in-country researchers, again applying the higher rates for outside researchers would have increased some elements by just over 3%. As explained in the Methods section, the inputs from overseas institutes in supervision and design were not included in the cost calculations. These inputs have been made in all the Tiny Target projects and have served to refine the technology as well as supporting the research component. Thus they will have contributed to the success of the operation. However, the cost analysis for Bonon, like the previous costs studies [29,30], was conceived as showing what it cost to implement a Tiny Target operation in the field by in-country experts.

The two years analysed were the first two years of the project operation, and included the initial deployment. Over a longer period, as mentioned above for deployment and monitoring, it may be that the time and people required to do the work can be slightly reduced. In fact, with the numerous rounds done, the increased familiarity with the location and greater experience and knowledge among the local people involved in the control work, made it possible to decrease the time spent in the field. Also, the maintenance of targets can be progressively transferred to CHWs. Thus, some cost reductions in future years would be expected. The Tiny Target work in Bonon is still ongoing, so the assumption made here, that the costs of the initial sensitisation and baseline T0 tsetse survey should be spread over only four years, was a conservative one and slightly overestimated costs, since the has work continued into its sixth year.

Another methodological point, to be considered for estimating the cost of future Tiny Target operations, is Vestergaard Ltd’s willingness to supply the ready-made insecticide-impregnated netting and fabric for targets at zero cost, whereas at the time this project was implemented the Tiny Targets used in Bonon were bought for USD 2.50 each. In terms of budgeting, this would reduce the financial costs of the operation analysed by 8%. However, in terms of an economic analysis of the resources used for the operation, its cost would remain unchanged.

The cost analysis for Côte d’Ivoire highlights some important underlying differences between the three Tiny Target operations whose costs were studied, as summarised in Table 9. The cost per km^2^ of gHAT focus protected by the tsetse control operation in Côte d’Ivoire was USD 471, as compared to USD 88 for Uganda (when updated to 2016 prices) and USD 67 for Chad. The higher cost in Côte d’Ivoire is due to a number of factors, which have general relevance for understanding the costs of tsetse control in different situations and for selecting metrics for comparing these costs in different settings.

Most straightforward is the use of a larger version of the Tiny Target, which measured 0.75m wide by 0.5m high as against 0.5m wide by 0.25m high for the targets used in Chad and Uganda. This reflected the different tsetse species (*G*. *p*. *palpalis* in Côte d’Ivoire as against *G*. *f*. *fuscipes* in Chad and Uganda) as well as the degraded forest biotope. Experimentation indicated that the larger target was more attractive for this species in this biotope [42]. These larger targets cost USD 2.5 for the finished insecticide-impregnated fabric component, but USD 3.7 when the fixings, freight, insurance and customs charges were added, as compared to USD 1.0 for the fabric and USD 1.6 in total for Chad and USD 1.4 in Uganda, where the purchase cost estimate was based on transport by sea freight. In addition, there were more targets per km^2^ protected: 14.9 in Côte d’Ivoire as against 6.2 in Uganda and 3.2 in Chad.

There were also some underlying differences in price levels. As can be seen from the international purchasing power parity comparisons between countries produced by the World Bank (https://data.worldbank.org/indicator/PA.NUS.PPPC.RF) in 2016 general prices levels in in Côte d’Ivoire were slightly higher than in Chad, and in both countries were about 20% higher than in Uganda. The main price difference, however, was linked to the organisational context in which the operations were conducted and was reflected in the high share of salaries in total costs. In Uganda the Tiny Target project was implemented almost entirely by local labour, and the costs of project supervision were based on the salary of a local district entomologist. In Côte d’Ivoire and Chad, the project was embedded in local research institutes, IPR and the Institut de Recherche en Élevage pour le Développement (IRED), respectively. Their staff did much of the project work, supported by labour recruited in the intervention area. In both countries, staff costs accounted for a high proportion of the Tiny Target costs.

The cost of ‘delivering’ a target was substantially higher in Bonon. If the total annual intervention costs are divided up between the cost of the targets, their shipping and their fixings (11.9% of total annual costs in Côte d’Ivoire, 8.3% in Chad and 15.3% in Uganda), then the cost of ‘delivering’, that is maintaining a target in place for a year, comes to USD 27.9 for Côte d’Ivoire as against USD 19.0 for Chad and USD 12.0 for Uganda. This ‘delivery’ cost thus includes everything except the cost of the targets: all other equipment, travel, staff and administrative costs for all activities and the full costs of all survey, monitoring and sensitisation work. Finding an appropriate comparator was more complicated for Uganda, where 61% of the targets were replaced during the maintenance round in the study year, and two full deployments are now undertaken [29,40]. The ‘delivery’ cost above was calculated on the basis of the 1550 targets in place at any one time, however, dividing the delivery costs by the total number of individual targets deployed (that is including the replacements made) reduces the delivery cost per target to USD 7.4. There were also differences between the operations in the way the tsetse monitoring was structured. In Uganda monitoring was done alongside other activities and thus throughout the second half of the year costed. In Chad, there were three annual field trips for monitoring, in Côte d’Ivoire, after the first year, there were four annual monitoring trips. Lastly, in Uganda, the project area was close to the nearby town of Arua, so that some of the work was done by bicycle or by motorbike. The Mandoul focus in Chad is an isolated area, and Bonon is also far away from the IPR headquarters, so that both required substantial travel to access them. These various factors contributed to the differences in the delivery costs. However, there are more important underlying reasons, which are explored below.

The four Tiny Target papers describing the control operations all include deployment maps. From these it becomes very clear that each area required a different approach to control tsetse. In Uganda the deployment was exclusively along the river banks [40]. In Chad, the targets were again along the both sides of a watercourse, but in this case the Mandoul River widened out into a swamp which in some locations was more than a kilometre across. Canoes were used to deploy targets and access sites [41]. The area where targets needed to be deployed was located only in the southwestern half of the gHAT focus, an area estimated at 45 km^2^ [41]. This differed from Uganda, where all rivers in the area had to be treated. The number of targets per km^2^ of protected area was thus lowest in Chad. In both countries the only vector is *G*. *f*. *fuscipes*. Although not covered by the cost studies, it is worth noting that the Tiny Target operation in Guinea was undertaken in yet another different type of tsetse habitat [39]. There, the vector is *G*. *p*. *gambiensis*, and the targets were placed along shores of the mangrove swamp inlets and islands, also along rivers in the savannah areas, but some targets were scattered inland. The target density in the protected area was much the same as in Côte d’Ivoire, 15 per km^2^. Lastly, in Bonon, although the targets were to some extent concentrated along rivers and in the low-lying areas, overall they were much more evenly spread throughout the 130 km^2^ protected area (Fig 1), because *G*. *p*. *palpalis* is widely distributed in the forest area of Côte d’Ivoire, being found even in towns or around the main villages [27,28].

Historically, publications on tsetse control were very explicit as to the area treated and area protected. The latter were also variously described as areas cleared or land made safe for grazing. This duality was routinely carried forward into calculations of cost [43]. The relationship between the area treated and the area protected varied according to tsetse species, control method used, tsetse habitat and climate. Looking at historical examples for West Africa, in northern Nigeria, where some 200,000 km^2^ were cleared of tsetse [17], the publications traced the need to treat a higher proportion of the land as the work moved southwards into more humid areas with denser vegetation. For groundspraying tsetse resting sites in the Lake Chad basin, to control *G*. *tachinoides*, *G*. *p*. *palpalis* and *G*. *m*. *submorsitans*, Davies [15,16] reported treating 12–20% of the land area in the northern areas, increasing to 50% further south. For helicopter spraying, it was stated “when 50% of the project area was situated in the southern Guinea savanna zone, the percentage of the reclaimed area sprayed rose from an average of 10–11% to almost 16%” [44]. Turning to the early examples of tsetse control in gHAT foci in Côte d’Ivoire, using insecticide-treated screens in Vavoua against *G*. *p*. *palpalis*, it was reported that just under 20% of the area protected was treated [22]. In Uganda, tackling *G*. *f*. *fuscipes* in an rHAT focus, the use of pyramidal traps is described, with an average density of 4 per km^2^ protected, but located in between 25% and 40% of the protected area [45,46]. Both these projects relied primarily on local farmers putting screens/traps in key locations in their farms or plantations. Lastly, two recent projects in West Africa cite the proportions of the protected area treated. In Ghana, using aerial spraying against *G*. *tachinoides* and *G*. *p*. *gambiensis*, 37% of the area was treated [47]. In Senegal, 23% of the area contained suitable habitat for the deployment of targets used for suppression of a *G*. *p*. *gambiensis* population [48].

In some cases it has been assumed that the area treated is equal to the area protected. This has been the case for some forms of aerial spraying [43]. Sometimes for areas containing only savannah (morsitans) group flies, the implicit assumption has tended to be that entire intervention area would be treated. In a series of economic analyses focussing on morsitans group tsetse, the only explicit distinction made between area treated and area reclaimed was in relation to locations where retreatment was required because some tsetse survived or tsetse reinvaded some of the area [49].

Overall, estimating the treated area is not straightforward, and the methods used are often inconsistent and not always clearly explained. Where treated areas are only measured as linear km, it becomes even more challenging to make comparisons. The relationship of the treated to the protected area is ultimately a function of the tsetse control technique deployed, together with the tsetse species involved and local ecology. Alongside these factors, the extent of area protected reflects the area’s bioecology and its human geography.

When estimating the protected area, there are several important considerations. From the entomological point of view, it has to include the furthest seasonal dispersal of tsetse flies. From the geographical point of view, it has to include people or livestock living in or travelling into the tsetse-infested area to work or graze, so as to map the populations at risk. For both gHAT and rHAT this can be done by tracing patients and by interviewing local populations about their movements [32,34,50]. A similar approach can be used for livestock populations, especially where these involve important seasonal shifts and the need to travel some distances to access water, especially during the dry season and in lower rainfall regions. These movement patterns will clearly vary both by local physical geography, people’s economic activities and, importantly, in relation to climate. Since the diversity of tsetse habitats and bioecology will vary depending on tsetse species, it will always lead to different deployment strategies. Thus, from the economic point of view, it is essential that the methodology for estimating populations (which could be animal as well as human) and area protected is clearly explained. Over time, working towards greater standardisation of the methods used for making these estimates would be an important goal.

In economics, cost-effectiveness can only be assessed in relation to a chosen measure of impact. Thus, Table 9 provides a range of comparators for each Tiny Target operation. The discussion above highlights the clear need to quantify and cite several indicators when assessing such tsetse control interventions. In areas where there are substantial livestock populations which are also affected by tsetse-transmitted trypanosomiasis, involving trypanosomes pathogenic to cattle and to other livestock species, there are likely to be spillover effects from tsetse control [30,41]. In some situations, especially where rHAT is present and the main reservoir is thought to be in cattle, tsetse control work is aimed at improving both animal and human health [51]. In Bonon, this type of ‘One Health’ impact is also expected to have taken place, given the substantial cattle and pig populations, the high decrease in tsetse population density and in infections in tsetse due to *Trypanosoma spp* found in this area [28,52].

However, ultimately, for a gHAT or rHAT focus, a key and well-established criterion is the annual cost per person protected [22,45]. In Chad’s Mandoul focus, with a population of about 39,000 this came to USD 1.4 per year. The Bonon focus in Côte d’Ivoire is estimated to contain 120,000 people, so that the cost per person protected comes to USD 0.5 per year. Within the area covered by the costed Tiny Target operation in Uganda, a full population census was not undertaken, but the population estimates point to an annual cost of about USD 0.2 per person protected. These figures can be compared with the estimated historic costs of other tsetse control operations in sleeping sickness foci, updated to 2016 prices [36] of USD 13 per person protected for Côte d’Ivoire (gHAT) [22] and USD 2 for Uganda (rHAT) [45]. In common with the other metrics the cost per person protected has its limitations. It does not reflect the current prevalence of gHAT, nor the number of new cases prevented in a particular year, even less vector control’s impact in preventing future gHAT epidemics. It is influenced by population density, which means more people are at risk of contracting gHAT, but this may not be reflected in the incidence of the disease, which will also be influenced by other control strategies historically or currently in place, notably passive and active detection. Commenting on the impacts on transmission and on current and future gHAT incidence are outside the scope of this paper, however, the impact of existing vector control activities on transmission has been modelled in Chad, the Democratic Republic of Congo (DRC) and Guinea e.g. [41,53–55] and health economic evaluations based on transmission model outputs have been able to investigate cost per disability-adjusted life year averted as vector interventions are added to medical interventions in the DRC [56] or for a range of different prevalence settings [57].

Thus, as Table 9 shows, the cost-effectiveness of different interventions in different tsetse geographical settings, with different human and biophysical geographies and organisational contexts, need to be assessed and compared using several metrics, each of which has its strengths and weaknesses. Those planning future interventions will thus need to consider not just the tsetse species they are dealing with and the organisations who will be implementing the work, but also where targets will need to be deployed (only along rivers, throughout a partially forested area), how the human and animal populations affected are distributed and their movements in and out of the tsetse-infested zones.

In terms of the range of impact metrics quantified, the Tiny Target operation in Bonon has thus been highly cost-effective, despite the challenges of a partially forested high rainfall habitat favourable to *G*. *p*. *palpalis*, so that that targets had to be deployed throughout the gHAT focus rather than just along rivers. The operation reduced the tsetse population by >95% in those places where tsetse bite humans [28]. The remaining active foci of gHAT are principally located in Africa’s forest regions [58]. Understanding the deployment patterns and target densities required for effective tsetse control in these forest areas and how these affect costs will be essential as work progresses.

## Supporting information

S1 Data Field trip cost spreadsheet for Bonon tsetse control work. (XLSX)
